# Surface-Engineered Amino-Graphene Oxide Aerogel Functionalized with Cyclodextrin for Desulfurization and Denitrogenation in Oil Refining

**DOI:** 10.3390/gels12010033

**Published:** 2025-12-30

**Authors:** Zunbin Duan, Huiming Zhang, Qiang Tong, Yanfang Li, He Bian, Guanglei Zhang

**Affiliations:** 1 National Engineering Research Center for Colloidal Materials and School of Chemistry and Chemical Engineering, Shandong University, Jinan 250100, China; zb.duan@sdu.edu.cn (Z.D.); lyf36876@163.com (Y.L.); 2 College of Chemical Engineering and Materials, Shandong University of Aeronautics, Binzhou 256603, China; zhanghuiming1982@163.com; 3 Department of Safety Engineering, Qingdao University of Technology, Qingdao 266520, China; tongqiang@stu.qut.edu.cn

**Keywords:** graphene oxide aerogel, cyclodextrin functionalization, fuel refining, selective removal

## Abstract

The selective removal of trace heteroatomic contaminants from fuel remains a critical challenge for clean combustion and refinery upgrading, particularly due to the chemical stability and structural similarity of sulfur- and nitrogen-containing aromatics. Herein, a surface-engineered graphene oxide aerogel functionalized with cyclodextrin (*β*-CD-CONH-GO) is developed via covalent grafting to introduce well-defined host–guest recognition sites within a porous framework. Spectroscopic and microscopic characterizations confirm successful functionalization, preserved aerogel morphology, and accessible hybrid interfaces. The removal process for monocyclic, bicyclic, and tricyclic impurities is governed by synergistic molecular inclusion within the cyclodextrin cavity, interfacial hydrogen bonding, and secondary confinement provided by the aerogel porosity. Thus, the *β*-CD-CONH-GO exhibits efficient adsorption toward representative bicyclic impurities, and the removal performance follows the order of indole > quinoline > benzothiophene. Kinetic analysis demonstrates pseudo-second-order adsorption behavior, indicating chemisorption dominated by cooperative host–guest recognition and hydrogen bonding. It possesses removal selectivity even in mixed systems containing structurally similar aliphatic and aromatic competitors and maintains > 95% efficiency after five regeneration cycles via ethanol extraction, confirming superb durability. This study demonstrates a feasible pathway to design adsorbents for deep fuel refining and highlights cyclodextrin-based graphene hybrid aerogels as promising candidates for separations.

## 1. Introduction

The increasing demand for cleaner fuels has prompted the development of technologies aimed at achieving ultra-deep desulfurization and denitrogenation of petroleum-derived fuels [1,2]. With growing environmental concerns regarding the emission of sulfur and nitrogen oxides during the combustion of fuels, stringent regulatory standards now require the sulfur content of fuels to be reduced to levels as low as 10 μg g^−1^. Traditional hydrodesulfurization (HDS) and hydrodenitrogenation (HDN) processes, while extensively used, face significant limitations [3,4,5,6]. These processes typically require high temperatures, pressures, and hydrogen consumption, all of which contribute to increased energy demand and undesirable by-products. Moreover, these techniques are often ineffective in removing refractory sulfur- and nitrogen-containing heterocycles, such as dibenzothiophene (DBT), 4,6-dimethyldibenzothiophene (4,6-DMDBT), quinoline, and indole, which possess robust C–S and C–N bonds [7]. The strong bond strength and steric hindrance of these compounds inhibit their accessibility to the active sites in hydrogenation catalysts, leading to incomplete removal even under severe conditions. Consequently, there is an urgent need for more efficient and selective non-hydrogenative methods for deep desulfurization and denitrogenation that can complement traditional hydrogenation technologies [8,9,10,11]. Among the various non-hydrogenative alternatives, adsorption-based processes have garnered considerable attention due to their mild operating conditions, lower energy requirements, and high specificity [12]. Adsorbents can selectively target specific molecular species, allowing for precise removal of contaminants such as sulfur and nitrogen heterocycles [13]. However, the development of adsorbent materials that combine high adsorption capacities, excellent selectivity, and the ability to be reused multiple times remains a challenge. Traditional adsorbents, such as activated carbon and silica, often exhibit limited selectivity and low efficiency for deeply refined oils due to their non-specific adsorption properties and limited active sites.

Cyclodextrins (CDs), a class of cyclic oligosaccharides, present a promising solution to this challenge [14,15,16,17,18]. With their unique molecular structure, which consists of a hydrophilic exterior and a hydrophobic interior cavity, CDs are capable of forming stable inclusion complexes with various molecules through host–guest interactions [19,20,21]. The selective inclusion of guest molecules into the cyclodextrin cavity relies on the complementary size, shape, and polarity of the guest, enabling CDs to act as highly specific adsorbents for sulfur- and nitrogen-containing organic compounds, such as those commonly found in fuel products [22]. However, the practical application of CDs is often hindered by their low surface area, poor dispersion in nonpolar media, and chemical instability under harsh conditions. To address these limitations, considerable research has been devoted to immobilizing CDs on high-surface-area materials to enhance their stability and dispersion, thereby improving their overall performance as adsorbents. Among these materials, graphene oxide (GO) has emerged as a particularly attractive support due to its high surface area, excellent mechanical properties, and the abundance of oxygenated functional groups on its surface, which can be easily modified to anchor functional molecules such as CDs [23,24,25,26,27]. GO-based composites offer the combined benefits of high surface area for efficient adsorption and the unique host–guest chemistry of CDs for selective removal of pollutants [28,29]. Moreover, GO’s excellent structural stability ensures that the composite retains its integrity during use, offering long-term reusability.

**Scheme 1:**
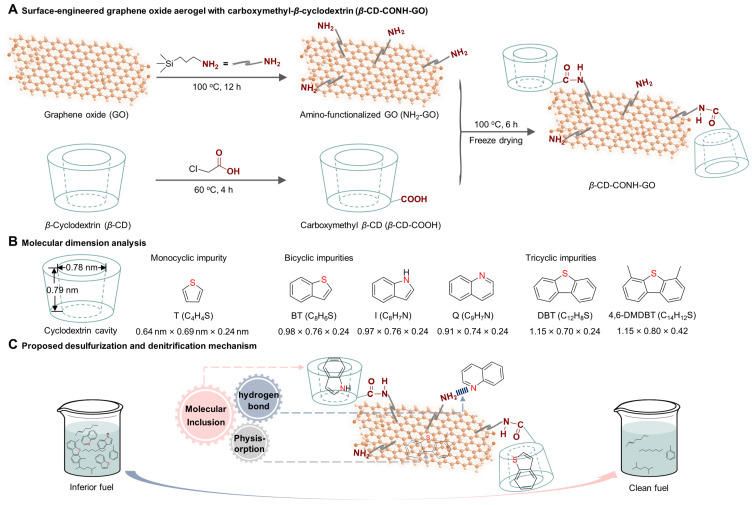
**Synthesis of *β*-CD-CONH-GO aerogel and its underlying mechanism of desulfurization and denitrogenation.** (**A**) Preparation procedure of *β*-CD-CONH-GO. (**B**) Sizes of cyclodextrin cavity and evaluated cyclic molecules showing appropriate matching between cyclodextrin and bicyclic impurities. (**C**) Proposed mechanism of desulfurization and denitrification using *β*-CD-CONH-GO.

Despite the appealing host–guest chemistry of CDs, straightforward mixing of CDs with GO often yields inconsistent desulfurization/denitrogenation performance because the CD distribution, interfacial stability, and, critically, the accessibility of the CD cavity are not well controlled [27,28,30]. Accordingly, this study focuses on the development of a surface-engineered amino-graphene oxide aerogel functionalized with carboxymethyl-*β*-cyclodextrin (*β*-CD-COOH). The material *β*-CD-CONH-GO combines the high surface area and structural stability of GO with the selective adsorption properties of *β*-CD-COOH. The rationale for selecting *β*-CD instead of *α*- or *γ*-CD is primarily based on the molecular size matching between the target sulfur- and nitrogen-containing heterocycles and the cavity dimensions of cyclodextrins [16,31,32,33]. The cavity size of *β*-CD provides the most suitable fit for the heterocyclic species investigated, whereas *α*-CD is too small and *γ*-CD is relatively oversized, leading to less effective host–guest interactions [16,34,35,36,37,38]. As shown in Scheme 1A, the *β*-CD-COOH molecules are covalently anchored onto amino-functionalized GO (NH_2_-GO) via amide linkages, ensuring the homogeneous dispersion of CDs while maintaining strong interfacial stability between the two components. This surface-engineered approach maximizes the availability of *β*-CD-COOH cavities for selective adsorption, making the freeze-dried *β*-CD-CONH-GO aerogel capable of efficiently removing thiophenic and nitrogen heterocyclic compounds with reasonable dimensions (Scheme 1B,C and Figure S1). The synergy between the high surface area of GO and the selective adsorption properties of *β*-CD-COOH opens avenues for the design of high-performance materials capable of addressing the challenges of ultra-deep desulfurization and denitrogenation in fuel processing. Unlike conventional CD-GO hybrids or CD polymers that are typically powder-like or densely packed [13,15,28], the aerogel architecture offers experimentally demonstrated advantages in terms of high permeability, interconnected porosity, and rapid diffusion. This study contributes to the growing field of sustainable fuel refining technologies and offers an approach to improving the environmental performance of petroleum-based fuels.

## 2. Results and Discussion

Graphene is oxidized into graphene oxide (GO) using a modified Hummers method, which contains a large number of functional groups on its surface, such as carboxyl, hydroxyl, and epoxy groups, making it highly reactive and amenable to bonding with organic molecules [25,39,40,41]. Initially, amino-functionalized GO (NH_2_-GO; post-functionalization) is synthesized by reacting the amino group of APTMS with the epoxy groups on the surface of GO. This reaction forms a stable amine linkage on the GO surface. Subsequently, carboxymethyl-*β*-cyclodextrin is chemically grafted onto the amino groups on GO via the carboxyl groups of *β*-CD-COOH, forming covalent bonds. This results in the formation of a stable and porous *β*-CD-CONH-GO aerogel with a density of 31.5 ± 2.8 mg cm^−3^ via freeze-drying (Figure S2A).

**Figure 1:**
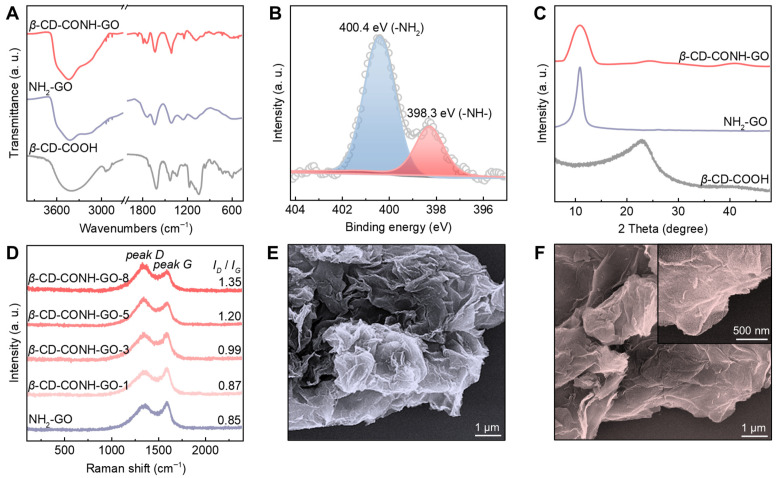
**Structural characterization of *β*-CD-CONH-GO aerogel.** (**A**) FT-IR spectra of *β*-CD-COOH, NH_2_-GO, and *β*-CD-CONH-GO. (**B**) High-resolution N 1s XPS spectrum of *β*-CD-CONH-GO. (**C**) XRD patterns of *β*-CD-COOH, NH_2_-GO, and *β*-CD-CONH-GO. (**D**) Raman spectra of NH_2_-GO and *β*-CD-CONH-GOs with different CD loadings. (**E**,**F**) SEM images of (**E**) NH_2_-GO and (**F**) *β*-CD-CONH-GO with a high magnification insert.

A series of spectroscopic and microscopic analyses are conducted to verify the successful fabrication and structural evolution of the cyclodextrin-modified graphene oxide aerogel. The FT-IR spectra of *β*-CD-COOH, NH_2_-GO, and the *β*-CD-CONH-GO aerogel reveal distinct characteristic bands (Figure 1A). The *β*-CD-COOH exhibits COO^−^ stretching bands at 1420 and 1605 cm^−1^, confirming carboxymethyl substitution [16]. After grafting APTMS, the NH_2_-GO shows absorption bands at 2940 and 2855 cm^−1^ assigned to asymmetric and symmetric –CH_2_– stretching, together with a peak at 1530 cm^−1^ corresponding to –NH_2_ bending vibration, indicating successful silane coupling. In the final *β*-CD-CONH-GO hybrid, a slight shift in C–N stretching from 1080 to 1070 cm^−1^, along with the emergence of *α*-glycosidic fingerprint vibration at 855 cm^−1^ confirms the formation of covalent amide linkages between *β*-CD-COOH and NH_2_-GO. Notably, the diagnostic amide signals (C=O stretching at ca. 1640–1680 cm^−1^ and N–H bending around 1550–1555 cm^−1^) cannot be unambiguously resolved because GO and CD contain multiple over-lapping oxygen-containing functionalities, and the newly formed amide content is relatively low, leading to weak/broadened bands. Therefore, high-resolution XPS N 1s provides the decisive evidence for covalent coupling (Figure 1B), where N 1s deconvolution displays binding energy components at 398.3 and 400.4 eV, corresponding to secondary amines (–N(H)–) and terminal amino groups (–NH_2_) [42], respectively, verifying covalent immobilization.

The powder X-ray diffraction (XRD) patterns of Figure 1C show that *β*-CD-COOH remains amorphous, while both NH_2_-GO and *β*-CD-CONH-GO display a diffraction peak at 2θ = 11.5°, characteristic of oxidized graphene. The absence of the 26° peak associated with graphite indicates complete oxidation during synthesis [22,30]. The reduced peak intensity and increased full width at half maximum in *β*-CD-CONH-GO suggest that cyclodextrin grafting partially disrupts ordered layer stacking, improving accessibility of adsorption interfaces. Raman spectra (Figure 1D) show typical D (ca. 1330 cm^−1^) and G (ca. 1590 cm^−1^) bands of carbon [25,43]. The increasing ratio of the D to G peak intensities (I_D_/I_G_) correlates with higher CD loading, reflecting the introduction of additional defect sites and reduced graphitic ordering [44,45], which is beneficial for enhancing surface interaction activity.

Scanning electron microscopy (SEM) images collected at different magnifications shown in Figure 1E,F and Figure S2B–D indicate that the NH_2_-GO exhibits wrinkled nanosheets with a relatively intact layered morphology. After cyclodextrin functionalization, the *β*-CD-CONH-GO shows retains a sheet-like structure but changed wrinkle density and surface roughness, consistent with structural disorder observed in Raman and XRD. Notably, no active cyclodextrin domains are observed, implying homogeneous distribution along the GO surface.

Nitrogen adsorption–desorption measurements (Table 1 and Figure S3) reveal that the specific surface area and pore volume gradually decrease with increasing CD content due to partial pore occupation. Among the four cyclodextrin functionalized GO samples, the *β*-CD-CONH-GO-5 exhibits the largest average pore diameter (2.75 nm), suggesting an optimized balance between accessible active sites and structural diffusion pathways within the aerogel network, which may translate to superb removal performance.

To assess the performance of the synthesized materials, representative sulfur- and nitrogen-containing model compounds are selected, including thiophene (T; C_4_H_4_S; CAS 110-02-1), benzothiophene (BT; C_8_H_6_S; CAS 95-15-8), dibenzothiophene (DBT; C_12_H_8_S; CAS 132-65-0), 4,6-dimethyl-dibenzothiophene (4,6-DMDBT; C_14_H_12_S; CAS 1207-12-1), quinoline (Q; C_9_H_7_N; CAS 91-22-5), and indole (I; C_8_H_7_N; CAS 120-72-9). As shown in Scheme 1B, the molecular dimensions of the bicyclic species (BT, Q, and I) are highly compatible with the hydrophobic cavity of cyclodextrin, implying that the *β*-CD-CONH-GO aerogel possesses structural selectivity favorable for their removal. All removal experiments are performed in triplicate, and the average removal efficiencies with errors within ±5.0% (representative result shown in Figure S4A) are used for analysis.

**Figure 2:**
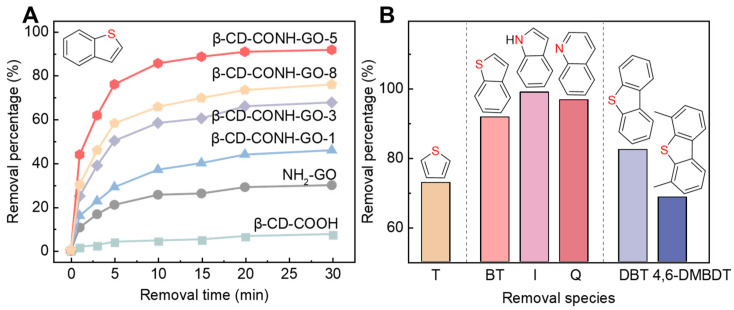
**Properties of *β*-CD-CONH-GO for removing sulfur and nitrogen compounds in simulated fuel.** (**A**) Removal efficiency of BT using synthetic *β*-CD-COOH, NH_2_-GO, and *β*-CD-CONH-GOs (operation parameters: 0.2 g of agent; 4.0 g of *n*-heptane containing 100 μg-S g^−1^; 30 °C). (**B**) Removal efficiency of T, BT, I, Q, DBT, and 4,6-DMDBT by *β*-CD-CONH-GO-5 (operation parameters: 0.2 g of agent; 4.0 g of *n*-heptane containing 100 μg-S or N g^−1^; 30 °C; 30 min).

The removal performance is first evaluated using BT (initial sulfur concentration, 100 μg g^−1^) dissolved in *n*-heptane with a sorbent-to-oil mass ratio of 1:20 at 30 °C (Figure 2A; Equation (S1)). The desulfurization performance increases with time and eventually reaches equilibrium, with the efficiency in the order of *β*-CD-CONH-GO-5 (91.8%) > *β*-CD-CONH-GO-8 (75.9%) > *β*-CD-CONH-GO-3 (67.7%) > *β*-CD-CONH-GO-1 (45.8%) > NH_2_-GO (29.8%) > *β*-CD-COOH (7.4%). Thus, the dispersed cyclodextrin critically governs adsorption behavior. As the cyclodextrin loading increases, the ability to remove BT improves up to an optimum at 5 m%. When the loading exceeds 5 m%, a slight decline in performance is observed, which may be attributed to excessive cyclodextrin causing steric hindrance or partial masking of the active cavities. At low loading of <5 m%, the reduced number of accessible cyclodextrin cavities limits host–guest interactions with the target molecules.

For *β*-CD-CONH-GO with the optimal loading of 5 m%, further desulfurization and denitrogenation experiments are conducted using other sulfur and nitrogen compounds. As shown in Figure 2B, the removal efficiency of the bicyclic compounds, including BT, Q, and I, is higher than that for monocyclic (T) or tricyclic (DBT and 4,6-DMDBT) species. The removal efficiencies of nitrides (Q and I) slightly exceed that of sulfide (BT), likely due to stronger hydrogen-bonding interactions between their nitrogen atoms and the hydroxyl groups of cyclodextrin. Importantly, the *β*-CD-CONH-GO demonstrates the capability to simultaneously remove both sulfur and nitrogen compounds from fuels, which is challenging for many conventional non-hydrogenative methods.

**Figure 3:**
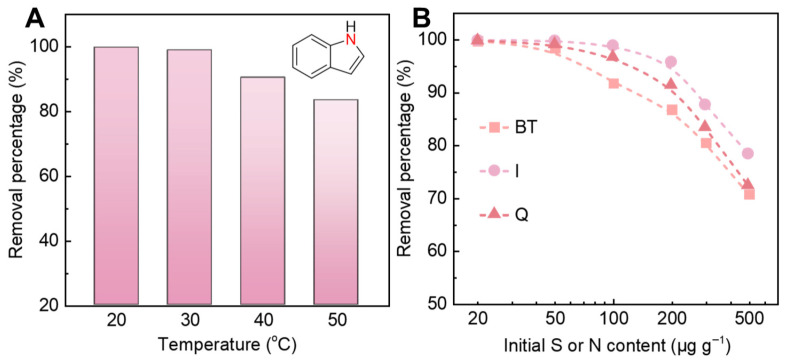
**Effect of (A) operating temperature and (B) initial heteroatom content on performance of *β*-CD-CONH-GO for removal of bicyclic impurities.** Removal parameters for (**A**) 0.2 g of agent; 4.0 g of *n*-heptane containing 100 μg-N g^−1^; 30 min; and (**B**) 0.2 g of agent; 4.0 g of *n*-heptane; 30 min; 30 °C.

The influence of operating temperature on the indole removal from *n*-heptane by *β*-CD-CONH-GO is investigated at 20, 30, 40, and 50 °C (Figure 3A). Within this range, the removal capacity decreases progressively with increasing temperature, indicating that the adsorption on *β*-CD-CONH-GO is an exothermic process. Consequently, ambient or mildly low-temperature conditions are favorable for maximizing performance while minimizing energy input.

The effect of initial heteroatom (sulfur or nitrogen) concentration on the performance of *β*-CD-CONH-GO is studied with the three bicyclic compounds, as shown in Figure 3B. When the initial sulfur or nitrogen content exceeds 100 μg g^−1^, the removal efficiency of BT, I, and Q declines slightly. At lower concentrations (<100 μg g^−1^), the *β*-CD-CONH-GO consistently achieves >90% of removal efficiency, indicating that after treatment, the residual sulfur or nitrogen levels is below the current regulatory limit of 10 μg g^−1^. It highlights the *β*-CD-CONH-GO’s potential for the refining of low-sulfur and low-nitrogen oil product.

**Figure 4:**
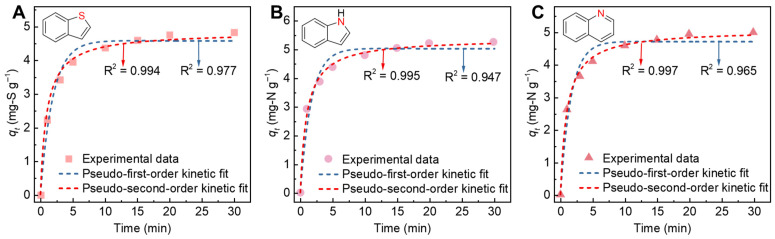
**Kinetic evaluation of removal process using *β*-CD-CONH-GO.** (**A**) BT. (**B**) I. (**C**) Q. Operation parameters: 0.2 g of agent; 4.0 g of *n*-heptane containing 300 μg-S or N g^−1^; 30 °C.

Kinetic studies on the removal of BT, I, and Q by *β*-CD-CONH-GO are conducted using pseudo-first-order and pseudo-second-order models (Equations (S2)–(S4)). Across all three systems, the adsorption behavior conforms much more closely to the pseudo-second-order model (Figure 4 and Figure S4B; R^2^ > 0.99), demonstrating that the adsorption rate is governed by chemisorption [46]. This observation further indicates that host–guest inclusion and hydrogen-bonding interactions within the cyclodextrin cavities dominate the adsorption process, whereas film diffusion and intraparticle diffusion contribute only marginally to the overall rate. The removal rate for the three bicyclic compounds follows the order of indole > quinoline > benzothiophene (Table S1). Indole exhibits the fastest kinetics with a rate constant of 0.207 g mg^−1^ min^−1^ (adsorption capacity, 5.38 mg-N g^−1^), likely due to its stronger hydrogen-bonding capability and higher electron density near the pyrrolic nitrogen, which enhances its affinity toward the hydroxyl groups and cavity of cyclodextrin. Quinoline (5.08 mg-N g^−1^) shows moderately high kinetics, while BT (4.84 mg-S g^−1^) displays the slowest uptake, which is consistent with the weaker interactions between sulfur-containing aromatics and cyclodextrin hydroxyl groups. The consistent kinetic trends among these heterocycles reinforce the mechanistic hypothesis that molecular polarity, heteroatom type, and cavity-fitting effects cooperatively control the adsorption behavior of *β*-CD-CONH-GO, and similar rankings are maintained at lower impurity levels (e.g., 100 μg g^−1^) used in performance evaluations.

**Figure 5:**
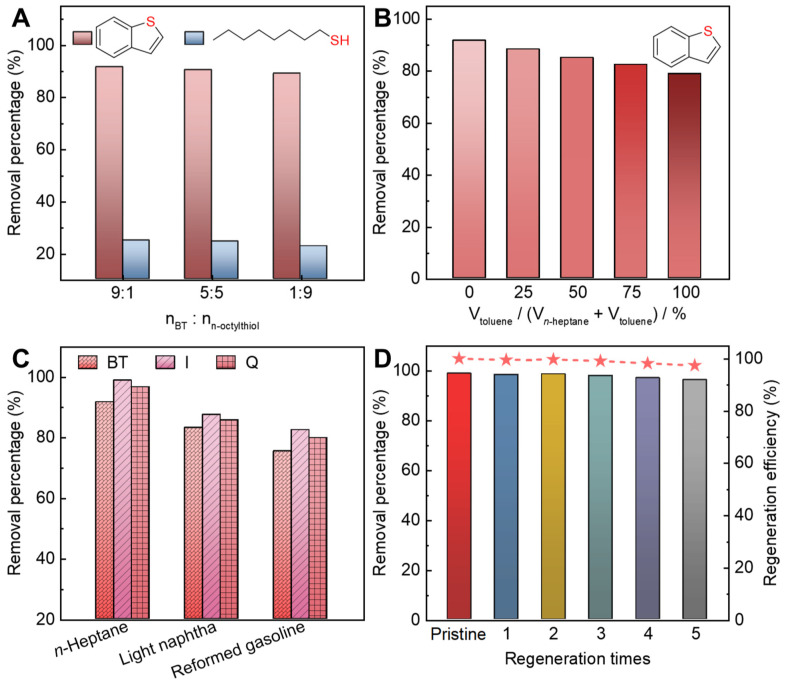
**Removal selectivity and regeneration performance of *β*-CD-CONH-GO.** (**A**) Selective removal of BT and *n*-octanethiol with three ratios in *n*-heptane. (**B**) Removal capacity of BT in *n*-heptane/toluene. (**C**) Removal properties for BT, I, and Q in light naphtha and reformate gasoline. (**D**) regeneration performance for removing indole from *n*-heptane, where the red star represents regeneration efficiency. Operation parameters: 0.2 g of agent; 4.0 g of fuel containing 100 μg-S or N g^−1^; 30 °C; 30 min.

Selectivity is an essential parameter when assessing the performance of *β*-CD-CONH-GO for fuel refining. To examine its selectivity, *n*-octanethiol (C_8_H_18_S; CAS 111-88-6), which has the same carbon number as BT but a distinctly different molecular geometry, is selected as a competitive sulfur species (Figure 5A). The *β*-CD-CONH-GO exhibits pronounced selectivity toward benzothiophene, achieving a removal efficiency of approximately 90% even when its initial sulfur concentration is as low as 10 μg g^−1^. This suggests that the rigid bicyclic structure of BT exhibits superb steric compatibility with the hydrophobic CD’s cavity, whereas the flexible alkyl chain of *n*-octanethiol results in a poor geometric fit, preventing the formation of stable inclusion complexes. This cavity-size-matching effect (Figure S1) is therefore the principal factor dictating the high selectivity of *β*-CD-CONH-GO.

To further probe the influence of molecular structure, the effect of aromatic and aliphatic hydrocarbons on removal behavior are investigated, as shown in Figure 5B,C. As the toluene content increases in mixed solvents, the removal efficiency of BT by *β*-CD-CONH-GO gradually decreases, indicating that aromatic compounds compete with BT for the cyclodextrin cavity. In two practical fuels (light naphtha containing mainly alkanes and cycloalkanes and reformate gasoline rich in aromatics; Figure S5), the *β*-CD-CONH-GO exhibits lower removal efficiency compared with *n*-heptane. Cycloalkanes and aromatics, owing to their cyclic geometry, show a tendency to interfere with the inclusion process. A rigorous, quantitative analysis of compositional changes and the extent to which competition depresses equilibrium capacity is not completed here due to experimental constraints and is targeted in future work.

The regeneration performance of *β*-CD-CONH-GO is evaluated using Soxhlet extraction with ethanol selected for its low toxicity, wide availability, and ease of recovery by distillation. Ethanol effectively disrupts the hydrogen-bonding and hydrophobic interactions stabilizing the inclusion complexes, thereby releasing the trapped heterocycles and restoring the active cyclodextrin cavities. After five consecutive regeneration cycles (Figure 5D), the *β*-CD-CONH-GO maintains a high removal efficiency toward indole, demonstrating superb structural stability, robustness, and recyclability. The minor decrease in removal capacity after multiple cycles maybe attributed to very little incomplete desorption. Regeneration under mild conditions, without high-temperature treatment or hydrogen input, underscores the potential energy-efficiency advantage of the proposed approach relative to conventional upgrading routes. In principle, the extracted heterocycles can be recovered from the regeneration solvent via established separation/purification steps (e.g., solvent recovery and fractionation) for potential reuse or downstream handling.

**Figure 6:**
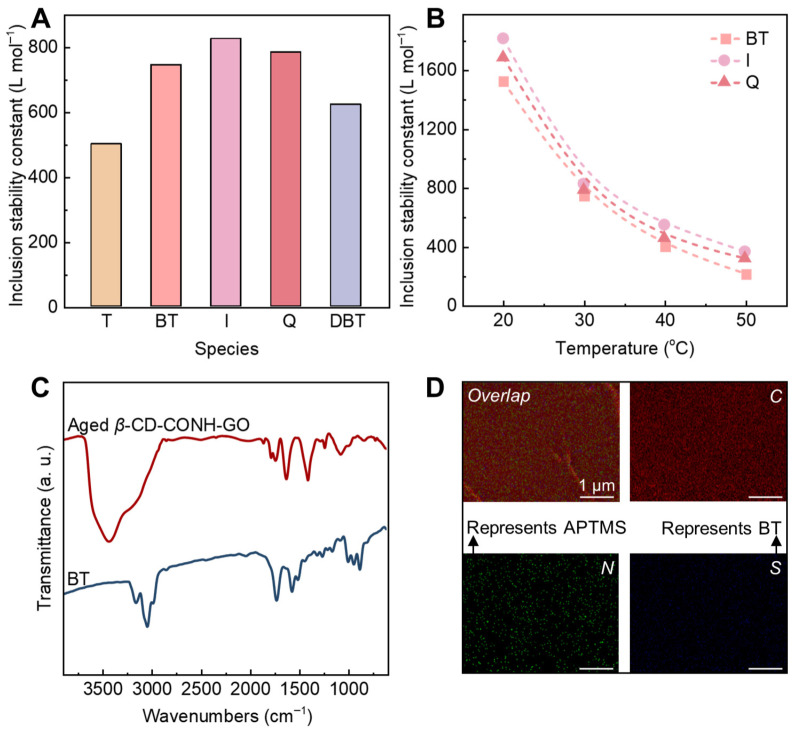
**Underlying mechanism of removal performance of *β*-CD-CONH-GO.** (**A**) Thermodynamic stability constants of inclusion complexes between *β*-CD-COOH and five studied compounds at 30 °C. (**B**) Inclusion stability constants of inclusion complexes between *β*-CD-COOH and bicyclic species at different temperatures. (**C**) FT-IR spectra of BT and aged *β*-CD-CONH-GO after removing BT. (**D**) Elemental mappings of C, N, and S for used *β*-CD-CONH-GO after removing BT.

To elucidate the removal mechanism, the host–guest interaction behavior between *β*-CD-COOH and representative sulfur- and nitrogen-containing heterocycles is first investigated using UV spectroscopy (Figure 6A,B). The cyclodextrin forms 1:1 stoichiometric inclusion complex with all tested heterocycles, confirming the formation of well-defined host–guest structures rather than nonspecific physical adsorption [13,14,20,47]. The corresponding thermodynamic stability constants reveal a trend: bicyclic molecules (BT, I, and Q) exhibit the highest affinity, followed by tricyclic species (DBT and 4,6-DMDBT), while monocyclic compound of T shows the weakest interaction. This hierarchy is fully consistent with the adsorption performance trend observed for *β*-CD-CONH-GO (Figure 2B), reinforcing that cyclodextrin cavities serve as the dominant active sites driving selective desulfurization and denitrogenation. Among the bicyclic compounds, the stability constant follows the order of indole > quinoline > benzothiophene. This trend correlates with molecular polarity and heteroatom type, and notably, this mechanistic trend matches the observed adsorption kinetics and equilibrium uptake, where I > Q > BT, demonstrating that inclusion strength fundamentally governs adsorption rate and capacity. Furthermore, the inclusion stability constants decrease gradually with increasing temperature, confirming the exothermic nature of the inclusion process. The reduced stability at elevated temperatures satisfactorily explains the temperature-dependent performance of *β*-CD-CONH-GO (Figure 3A). This thermodynamic behavior also supports the material’s suitability for low-energy, mild-operation purification scenarios.

To further verify the inclusion-dominant mechanism, spectroscopic analysis and microstructural mapping are performed using benzothiophene as a representative probe molecule. After adsorption, the FT-IR spectrum of *β*-CD-CONH-GO shows no distinguishable BT characteristic peaks (Figure 6C), indicating that the adsorbed species are included within the cyclodextrin cavities rather than exposed on the material surface. Complementary SEM elemental mapping shown in Figure 6D and Figure S6 reveals only trace sulfur signals distributed within the matrix rather than localized on the external surface, strongly suggesting that sulfur compounds diffuse into and reside within the cyclodextrin cavities. Given the even higher inclusion constants of nitrogen heterocycles, the same behavior is expected for Q and I.

Based on these analyses, the proposed solid–liquid desulfurization and denitrogenation mechanism of *β*-CD-CONH-GO relies on the synergistic multi-interaction system consisting of (1) cyclodextrin cavity-driven host–guest inclusion, (2) hydrogen bonding between heteroatoms and cyclodextrin hydroxyl groups, and (3) supplementary physical adsorption contributed by the graphene oxide aerogel framework (Scheme 1C). According to a comprehensive evaluation of the experimental results, including inclusion constant analysis, adsorption selectivity, kinetic behavior, and comparative performance trends, molecular inclusion is identified as the dominant mechanism, followed by hydrogen bonding, while confinement effects play a secondary role. During the removal process, the sulfur and nitrogen heterocycles preferentially enter the hydrophobic cavity of the cyclodextrin, forming stable inclusion complexes rather than relying solely on surface adsorption. Bicyclic molecules, owing to their rigid geometry and excellent cavity-fitting characteristics, demonstrate superior adsorption efficiency, which aligns with both thermodynamic and kinetic analyses. Additionally, nitrogen-containing molecules exhibit enhanced interaction through secondary hydrogen bonding, accelerating their removal relative to sulfur-containing analogues. This interaction mechanism explains the high selectivity, fast kinetics, and strong regeneration capability exhibited by *β*-CD-CONH-GO, which surpasses most of the reported adsorbents for BT (Table S2). More broadly, the aerogel architecture offers a practical route to translate cyclodextrin recognition chemistry into a permeable, reusable solid for deep fuel purification.

## 3. Conclusions

The *β*-CD-CONH-GO is established as a cyclodextrin-functionalized graphene oxide aerogel that integrates molecular recognition with a permeable porous scaffold for deep fuel purification. Covalent immobilization of *β*-CD-COOH on NH_2_-GO, followed by freeze-drying, yields a lightweight monolith with preserved aerogel morphology and accessible hybrid interfaces. Systematic loading studies identify *β*-CD-CONH-GO-5 as an optimal composition, balancing cavity availability with pore accessibility. Under mild conditions, the aerogel displays selective removal toward bicyclic heteroaromatics and achieves a consistent performance hierarchy (indole > quinoline > benzothiophene), while monocyclic and sterically hindered tricyclic species are removed less efficiently. Pseudo-second-order kinetics and inclusion-constant analyses collectively indicate that adsorption is governed primarily by cyclodextrin-cavity inclusion, reinforced by hydrogen bonding (especially for N-heterocycles) and assisted by framework confinement/physical adsorption. Competitive aromatics in mixed solvents and real fuels reduce removal efficiency, highlighting the practical importance of matrix effects in refinery streams. Notwithstanding, *β*-CD-CONH-GO is readily regenerated by ethanol extraction and maintains high efficiency over multiple cycles, demonstrating durability under low-energy operation. Overall, this work provides a design paradigm for translating cyclodextrin host–guest chemistry into robust graphene-based aerogels and points to a scalable route for selective, regenerable adsorbents for ultra-deep desulfurization and denitrogenation.

## 4. Materials and Methods

The detailed experimental procedures can be seen in the Supplementary Materials, with only a brief description given in this part. The following chemical reagents were purchased from Shanghai Aladdin Biochemical Technology Co., Ltd. (Shanghai, China).

The synthesis of *β*-CD-CONH-GO proceeded through three sequential steps as follows: First, *β*-CD-COOH was synthesized through a nucleophilic substitution reaction between *β*-CD and monochloroacetic acid in an alkaline medium. 1 mmol of *β*-CD was introduced into a 100 mL flask equipped with a reflux condenser, and 2 mL of deionized water was added. The mixture was stirred for 10 min, followed by the addition of sodium hydroxide solution to adjust the pH to 12. The solution was heated to 60 °C and stirred until *β*-CD was completely dissolved, and monochloroacetic acid was then added dropwise over 20 min. The mixture was heated at 60 °C for 4 h. Upon completion, the pH of the solution was adjusted to 7 using acetic acid, and the solution was cooled in a dark place for 12 h to complete the reaction. The mixture was precipitated by adding a 1:1 mixture of anhydrous ethanol and acetone, followed by standing for 0.5 h and separation of the precipitate. The product was then dissolved in minimal deionized water, washed three times, and vacuum-dried at 60 °C for 6 h. The yield of *β*-CD-COOH was 53%, with a substitution degree of 2.8 determined by non-aqueous chemical titration. Second, GO was prepared using a modified Hummers method (KMnO_4_ oxidation in acidic medium). 0.50 g of GO was added to a 500 mL flask, and 200.0 mL of toluene was introduced. The mixture was sonicated under nitrogen protection for 1 h to disperse and prepare the dispersion. Subsequently, 5.0 mL of 3-aminopropyltrimethoxysilane was added in one shot, and the reaction was carried out at 100 °C under nitrogen for 12 h. After cooling to room temperature, the reaction mixture was filtered under vacuum, and the resulting filter cake was washed three times with ethanol and acetonitrile to remove any unreacted materials. The solid was then dried under vacuum to yield the NH_2_-GO. The *β*-CD-CONH-GO hybrid was synthesized by covalently bonding *β*-CD-COOH to NH_2_-GO. Typically, 100 mg of NH_2_-GO was mixed with 5.0 mg of *β*-CD-COOH in 20.0 mL of *N*-methylpyrrolidone under nitrogen. The mixture was sonicated for 1 h to ensure uniform dispersion. The reaction was then carried out at 100 °C for 6 h. Afterward, the mixture was cooled to room temperature, and the product was filtered under vacuum. The resulting filter cake was washed with 25 mL of ethanol three times, and then freeze-dried to obtain a 92 mg of the hybrid product, labeled as *β*-CD-CONH-GO. The theoretical *β*-CD-COOH loading in the hybrid was 5% (*β*-CD-CONH-GO-5). Similarly, other samples with *β*-CD-COOH loadings of 1%, 3%, and 8% were prepared, resulting in *β*-CD-CONH-GO-1, *β*-CD-CONH-GO-3, and *β*-CD-CONH-GO-8, respectively.

Performance of *β*-CD-CONH-GO for removal of sulfur and nitrogen compounds was carried out under mild temperatures (20–50 °C). The simulated fuel (*n*-heptane) containing sulfur or nitrogen impurity was prepared, and the *β*-CD-CONH-GO was dried at 70 °C for 1 h before use. The simulated fuel and *β*-CD-CONH-GO were mixed in a mass ratio of 20:1 (*β*-CD-CONH-GO, 0.2 g) under stirring (300 rpm). The sulfur or nitrogen content at different times was estimated using an ANTEK 9000NS analyzer (Antek Instruments, Houston, TX, USA; total sulfur/total nitrogen determination). All the evaluation experiments were performed three times, and the average removal efficiency (error ≤ 5.0%) was used for analysis. The regeneration of the aged *β*-CD-CONH-GO was achieved through Soxhlet extraction using cheap polar solvent of ethanol. The aged *β*-CD-CONH-GO was extracted 3 times with ca. 70 mL of ethanol in a 50 mL Soxhlet extractor to complete regenerations. The used ethanol (approximately 50 mL per regeneration cycle) was recovered by distillation and reused in subsequent cycles, minimizing solvent consumption and waste generation at the laboratory scale.

## Figures and Tables

**Table 1:** Textural properties of NH_2_-GO and *β*-CD-CONH-GOs.

Samples	BET Surface Area (m^2^ g^−1^)	Pore Diameter (nm)	Volume Pores (cm^3^ g^−1^)
NH_2_-GO	1098.6	2.89	0.74
*β*-CD-CONH-GO-1	954.6	2.68	0.67
*β*-CD-CONH-GO-3	944.1	2.72	0.62
*β*-CD-CONH-GO-5	926.7	2.75	0.59
*β*-CD-CONH-GO-8	904.6	2.71	0.57

## Data Availability

The original contributions presented in this study are included in the article/Supplementary Materials. Further inquiries can be directed to the corresponding authors.

## Supplementary Materials

The following supporting information can be downloaded at: https://www.mdpi.com/article/10.3390/gels12010033/s1, experimental procedures; Figure S1: Structures and sizes of cyclodextrin cavity and compounds used; Figure S2: Macroscopic photos and microscopic morphology of *β*-CD-CONH-GO; Figure S3: N_2_ adsorption and desorption isotherms and pore distributions of NH_2_-GO and *β*-CD-CONH-GOs; Figure S4: Removal efficiency of BT with error bars and removal kinetic evaluation using *β*-CD-CONH-GO at an initial sulfur content of 100 μg g^−1^; Figure S5: Hydrocarbon compositions of light naphtha and reforming gasoline; Figure S6: SEM-mapping images of C, O, N, and S elements in aged *β*-CD-CONH-GO after removing BT; Table S1: Kinetic fitting parameters of bicyclic-impurity removal process using *β*-CD-CONH-GO; Table S2: Comparisons of *β*-CD-CONH-GO and reported agents for removal of BT sulfur. References [13,15,22,28,48,49,50,51,52] is cited in the Supplementary Materials.
