# Supplementary material for: Surface-Engineered Amino-Graphene Oxide Aerogel Functionalized with Cyclodextrin for Desulfurization and Denitrogenation in Oil Refining
Source: Gels. 2025 Dec 30;12(1):33. doi: 10.3390/gels12010033 (PMC12840899; doi:10.3390/gels12010033)
Supplement: Supplementary file 1 [file gels-12-00033-s001.zip › gels-4056452-supplementary.pdf]

gels

MDPI

Supplementary Materials for

Surface-Engineered Amino-Graphene Oxide Aerogel Functionalized with Cyclodextrin for Desulfurization and Denitrogenation in Oil Refining

Zunbin Duan [1], Huiming Zhang [2], Qiang Tong [3], Yanfang Li [1], He Bian [2,*] and Guanglei Zhang [1,*]

[1] National Engineering Research Center for Colloidal Materials and School of Chemistry and Chemical Engineering, Shandong University, Jinan 250100, China
[2] College of Chemical Engineering and Materials, Shandong University of Aeronautics, Binzhou 256603, China
[3] Department of Safety Engineering, Qingdao University of Technology, Qingdao 266520, China
* Correspondence: hbiansn@163.com (H.B.); zhanggl@sdu.edu.cn (G.Z.)

SECTION S1. Experimental Procedures

S1.1. Materials

Graphite powder (CAS 7782-42-5; ≥99.95% metals basis), *β*-cyclodextrin (*β*-CD; $C_{42}H_{70}O_{35}$; CAS 7585-39-9; ≥98%), 3-aminopropyltrimethoxysilane (APTMS; $C_6H_{17}NO_3Si$; CAS 13822-56-5; ≥97%), monochloroacetic acid ($C_2H_3ClO_2$; CAS 79-11-8; ≥99%), thiophene (T; C_4H_4S; CAS 110-02-1; ≥99%), benzo[b]thiophene (BT; C_8H_6S; CAS 95-15-8; ≥97%), dibenzothiophene (DBT; $C_{12}H_8S$; CAS 132-65-0; ≥99%), 4,6-dimethyldibenzothiophene (4,6-DMDBT; $C_{14}H_{12}S$; CAS 1207-12-1; ≥97%), indole (I; C_8H_7N; CAS 120-72-9; ≥99%), quinoline (Q; C_9H_7N; CAS 91-22-5; ≥98%), *n*-octanethiol ($C_8H_{18}S$; CAS 111-88-6; ≥98%), toluene, sodium hydroxide, and *N*-methylpyrrolidone were purchased from Shanghai Aladdin Biochemical Technology Co., Ltd (Shanghai, China). All chemical reagents were used as received without further purification.

S1.2. Synthesis of Cyclodextrin-Functionalized Graphene Oxide Hybrid

Synthesis of carboxymethyl-β-cyclodextrin (β-CD-COOH). *β*-CD-COOH was synthesized through a nucleophilic substitution reaction between *β*-cyclodextrin and monochloroacetic acid in an alkaline medium. 1 mmol of *β*-CD was introduced into a 100 mL three-neck round-bottom flask equipped with a reflux condenser, and 2 mL of deionized water was added. The mixture was stirred for 10 minutes, followed by the addition of sodium hydroxide solution to adjust the pH to 12. The solution was heated to 60 °C and stirred until *β*-CD was completely dissolved, and monochloroacetic acid was then added dropwise over 20 minutes. The mixture was heated at 60 °C for 4 hours. Upon completion, the pH of the solution was adjusted to 7 using acetic acid, and the solution was cooled in a dark place for 12 hours to complete the reaction. The mixture was precipitated by adding a 1:1 mixture of anhydrous ethanol and acetone, followed by standing for 30 minutes and separation of the precipitate. The product was then dissolved in minimal deionized water, washed three times, and vacuum-dried at 60 °C for 6 hours. The yield of *β*-CD-COOH was 53%, with a substitution degree of 2.8 determined by non-aqueous chemical titration.

Synthesis of amino-functionalized graphene oxide (NH_2-GO). Graphene oxide (GO) was prepared using a modified Hummers method ($KMnO_4$ oxidation in acidic medium). The obtained GO was subsequently dispersed in anhydrous toluene for APTMS grafting to produce NH_2-GO. 0.50 g of GO was added to a 500 mL three-neck round-bottom flask, and 200.0 mL of toluene was introduced. The mixture was sonicated under nitrogen protection for 60 minutes to disperse and prepare the dispersion. Subsequently, 5.0 mL of 3-aminopropyltrimethoxysilane was added in one shot, and the reaction was carried out at 100 °C under nitrogen for 12 hours. After cooling to room temperature, the reaction mixture was filtered under vacuum, and the resulting filter cake was washed three times with ethanol and acetonitrile to remove any unreacted materials. The solid was then dried under vacuum to yield the amino-functionalized graphene oxide (NH_2-GO).

Synthesis of cyclodextrin-functionalized graphene oxide (β-CD-CONH-GO) hybrid. The *β*-CD-CONH-GO hybrid was synthesized by covalently bonding carboxymethyl-*β*-cyclodextrin to the amino-functionalized graphene oxide. Typically, 100 mg of NH_2-GO was mixed with 5.0 mg of *β*-CD-COOH in 20.0 mL of *N*-methylpyrrolidone under nitrogen. The mixture was sonicated for 60 minutes to ensure uniform dispersion. The reaction was then carried out at 100 °C for 6 hours. Afterward, the mixture was cooled to room temperature, and the product was filtered under vacuum. The resulting filter cake was washed with 25 mL of ethanol three times, and then freeze-dried to obtain a 92 mg of

the hybrid product, labeled as β-CD-CONH-GO. The theoretical β-CD-COOH loading in the hybrid was 5% (β-CD-CONH-GO-5). Similarly, other samples with β-CD-COOH loadings of 1%, 3%, and 8% were prepared, resulting in β-CD-CONH-GO-1, β-CD-CONH-GO-3, and β-CD-CONH-GO-8, respectively.

S1.3. Materials Characterization

Scanning electron microscopy (SEM) was carried out on a Zeiss Supra 55 Sapphire field-emission scanning electron microscope (Jena, Germany) at 2.0 kV and a distance of 5.0 mm. Powder X-ray diffraction (XRD) was performed on a Rigaku SmartLab diffractometer (Rigaku Corporation, Japan) by using Cu Kα radiation (40 kV, 30 mA). X-ray photoelectron spectroscopy (XPS) was carried out on a Thermo Fisher ESCALab 250Xi spectrometer (Thermo Fisher Scientific, Waltham, Massachusetts, USA) with Al Kα radiation, and the obtained spectra were calibrated based on the C *1s* peak at 284.6 eV. The Brunauer-Emmett-Teller measurements were recorded with a Micromeritics ASAP 2020 surface area and porosimetry analyzer (Nocross, Georgia, USA). Raman scattering spectra were acquired on a Horiba Jobin-Yvon Lab Ram HR VIS confocal Raman microscope (Paris, France) at RT (excitation source, 633 nm laser). Fourier transform infrared (FT-IR) spectra were obtained from a Bruker Tensor II FT-IR spectrometer (Karlsruhe, Germany; KBr pellet method; scan number, 32).

S1.4. Removal Performance Evaluation

Performance of β-CD-CONH-GO for removal of sulfur and nitrogen compounds was carried out under mild temperatures (20-50 °C). The simulated fuel (*n*-heptane) containing sulfur or nitrogen impurity was prepared, and the β-CD-CONH-GO was dried at 70 °C for 1 h before use. The simulated fuel and β-CD-CONH-GO were mixed in a mass ratio of 20:1 (β-CD-CONH-GO, 0.2 g) under stirring (300 rpm). The sulfur or nitrogen content at different times was estimated using an ANTEK 9000NS analyzer (Antek Instruments, Houston, Texas, USA; total sulfur/total nitrogen determination). All the evaluation experiments were performed three times, and the average removal efficiency (error ≤ 5.0%) was used for analysis. The removal performance of β-CD-CONH-GO was calculated by the equations S1 and S2,

$$\text{Removal percentage (\%)} = (C_0 - C_t) / C_0 \times 100 \,, \quad \text{(S1)}$$

$$q_t = (C_0 - C_t)\, W / 1000M \,, \quad \text{(S2)}$$

where C_0 (μg g^{-1}) and C_t (μg g^{-1}) are the initial and remaining sulfur or nitrogen content at time *t* of the simulated oil, q_t (mg g^{-1}) is the sulfur or nitrogen capacity at time *t* (min), *W* (g) is the mass of the fuel, and *M* (g) is the mass of β-CD-CONH-GO, respectively.

The removal kinetic behaviors of β-CD-CONH-GO were fitted with a pseudo-first-order kinetic model (Eq. S3) and a pseudo-second-order kinetic model (Eq. S4),

$$q_t = q_e\,(1 - e^{k1t}) \,, \quad \text{(S3)}$$

$$q_t = k_2\, q_e^2\, t / (1 + k_2\, q_e\, t) \,, \quad \text{(S4)}$$

where q_e (mg g^{-1}) and q_t (mg g^{-1}) are the sulfur or nitrogen capacity at equilibrium and time *t* (min), and k_1 (min^{-1}) and k_2 (g mg^{-1} min^{-1}) are the kinetic rate constant of pseudo-first-order kinetic and pseudo-second-order kinetic model equations, respectively.

The regeneration of the used β-CD-CONH-GO was achieved through Soxhlet extraction using cheap polar solvent of ethanol. The used β-CD-CONH-GO was extracted 3 times with ca. 70 mL of ethanol in a 50 mL Soxhlet extractor to complete regenerations. The used ethanol was recovered by distillation.

S1.5. Calculation Simulations

https://doi.org/10.3390/gels12010033

The molecular sizes of T, BT, DBT, 4, 6-DMDBT, I, and Q was as follows. The molecule structure was optimized using the GFN-xTB method [48] on the xtb program developed by Grimme, and the three-dimensional size of the molecule was then measured through the Multiwfn software (Beijing, China).

https://doi.org/10.3390/gels12010033

SECTION S2. Supplementary figures

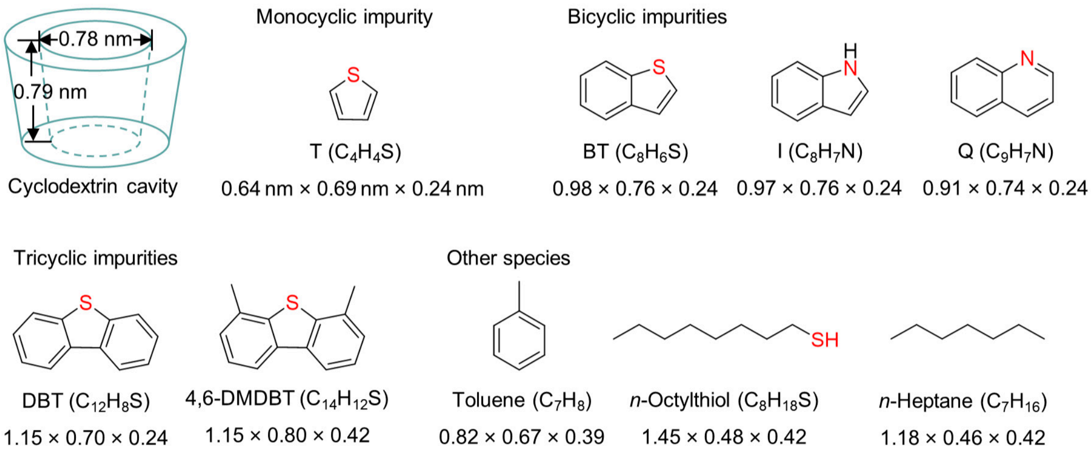

Figure S1. Structures and sizes of cyclodextrin cavity and compounds used.

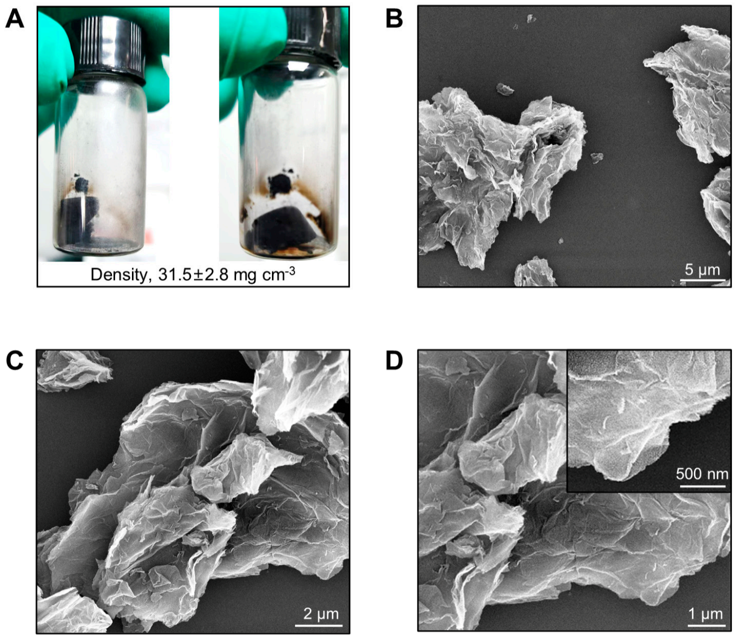

Figure S2. Macroscopic photos and microscopic morphology of *β*-CD-CONH-GO. (A) White light photograph of *β*-CD-CONH-GO with measured density of 31.5±2.8 mg cm^{-3}. (B-D) SEM images of *β*-CD-CONH-GO at different magnifications. The less pronounced 3D network appearance in some regions may be related to the sample preparation process (*e.g.*, fracture and mounting), which can expose layered edges and partially obscure the continuous network features.

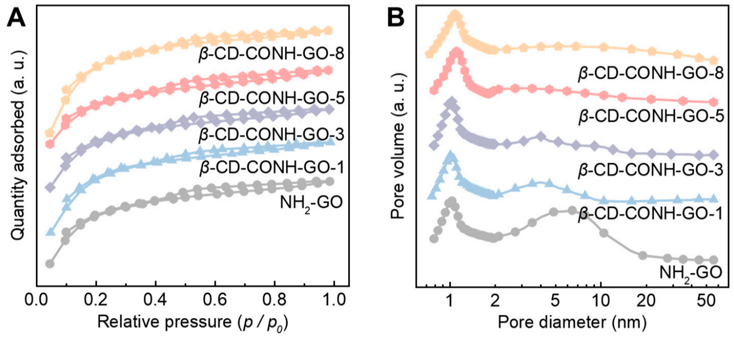

Figure S3. (A) N_2 adsorption and desorption isotherms and (B) pore distributions of NH_2-GO and *β*-CD-CONH-GOs.

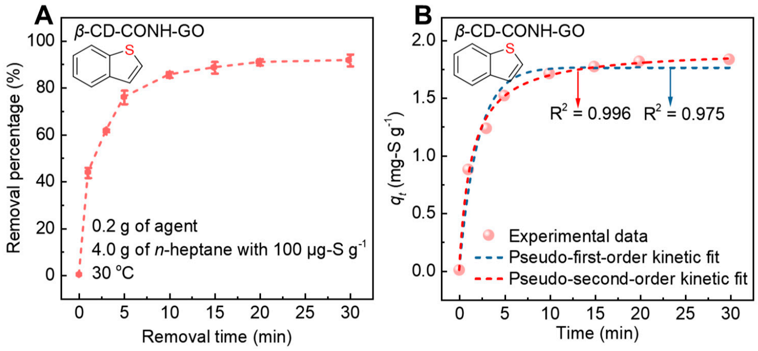

Figure S4. (A) Removal efficiency of BT with error bars and (B) removal kinetic evaluation using *β*-CD-CONH-GO at an initial sulfur content of 100 μg g^{-1}.

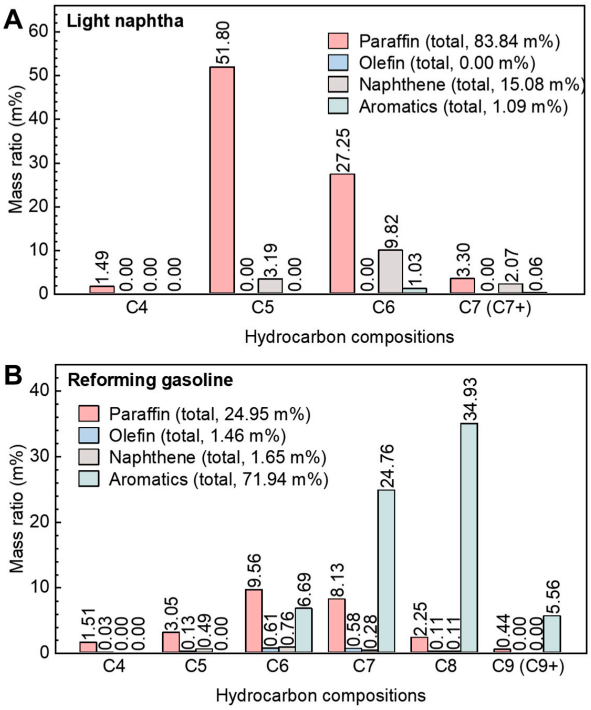

Figure S5. Hydrocarbon compositions of (A) light naphtha and (B) reforming gasoline.

https://doi.org/10.3390/gels12010033

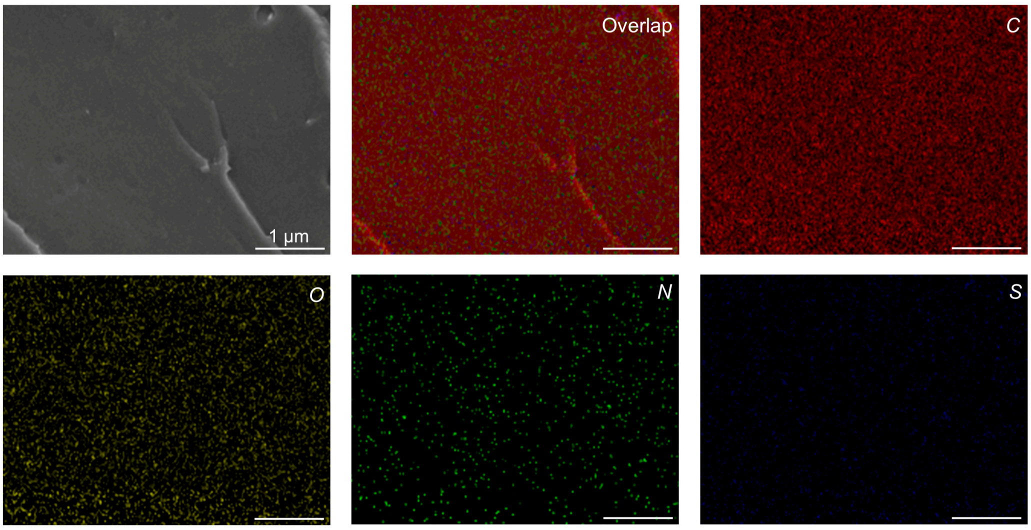

Figure S6. SEM-mapping images of C, O, N, and S elements in aged *β*-CD-CONH-GO after removing BT.

SECTION S3. Supplementary tables

Table S1. Kinetic fitting parameters of bicyclic-impurity removal process using *β*-CD-CONH-GO.

Impurity	Pseudo-first-order kinetic model			Pseudo-second-order kinetic model		
	q_e / mg g^{-1}	k_1 / min^{-1}	R^2	q_e / mg g^{-1}	k_2 / g mg^{-1} min^{-1}	R^2
BT	4.58	0.52	0.977	4.84	0.186	0.994
I	5.04	0.59	0.947	5.38	0.207	0.995
Q	4.72	0.61	0.965	5.08	0.197	0.997

https://doi.org/10.3390/gels12010033

Table S2. Comparisons of β-CD-CONH-GO and reported agents for removal of BT sulfur.

Agent	S_0 / μg g^{-1}	T / °C	M_{agent}:M_{fuel}	t / h	Efficiency / %	Ref.
β-CD-CONH-GO	100	30	1:20	0.5	92	This work
2-HP-β-CDP	100	30	1:20	1.5	91	[13]
6-HP-β-CDP	100	30	1:20	1.5	61	
2-HP-γ-CDP	100	30	1:20	2	79	[15]
β-CDP	100	25	1:20	2	50	[49]
α-CDP	100	25	1:20	2	25	
γ-CDP	100	25	1:20	2	28	
CD@SiO_2@Fe_3O_4	100	30	1:20	2	70	[22]
CD-CuO/NH_2-GO	100	30	1:20	1.5	73	[28]
MOF-5@AC	100	30	1:30	4	42	[50]
14-N-GR	300	70	1:150	2	93	[51]
MOF-74@γ-Al_2O_3	35	30	1:40	2	88	[52]

https://doi.org/10.3390/gels12010033

SECTION S4. Supplementary references

13. Duan, Z.; Bian, H.; Zhu, L.; Xia, D. Efficient removal of thiophenic sulfides from fuel by micro-mesoporous 2-hydroxypropyl-β-cyclodextrin polymers through synergistic effect. Sep. Purif. Technol. 2022, 300, 121884.
15. Duan, Z.; Wei, S.; Bian, H.; Guan, C.; Zhu, L.; Xia, D. Inclusion as an efficient purification method for specific removal of tricyclic organic sulfur/nitrogen pollutants in fuel and effluent with cyclodextrin polymers. Sep. Purif. Technol. 2021, 254, 117643.
22. Duan, Z.; Ding, X.; Wang, Y.; Zhu, L.; Xia, D. A new strategy for fuel desulfurization by molecular inclusion with cop-per(II)-β-cyclodextrin@SiO2 @Fe3O4 for removing thiophenic sulfides. Energy Fuels 2018, 32, 11421–11431.
28. Duan, Z.; Zhang, M.; Bian, H.; Wang, Y.; Zhu, L.; Xiang, Y.; Xia, D. Copper(II)-β-cyclodextrin and CuO functionalized gra-phene oxide composite for fast removal of thiophenic sulfides with high efficiency. Carbohydr. Polym. 2020, 228, 115385.
48. Bannwarth, C.; Ehlert, S.; Grimme, S. GFN2-xTB—An accurate and broadly parametrized self-consistent tight-binding quantum chemical method with multipole electrostatics and density-dependent dispersion contributions. *J. Chem. Theory Comput.* **2019**, *15*, 1652–1671.
49. Li, L.; Duan, Z.; Chen, J.; Zhou, Y.; Zhu, L.; Xiang, Y.; Xia, D. Molecular recognition with cyclodextrin polymer: A novel method for removing sulfides efficiently. *RSC Adv.* **2017**, 7, 38902-38910.
50. Zhu, L.; Jia, X.; Bian, H.; Huo, T.; Duan, Z.; Xiang, Y.; Xia, D. Structure and adsorptive desulfurization performance of the composite material MOF-5@AC. *New J. Chem.* **2018**, *42*, 384-385.
51. Khalse, N. M.; De, M. Adsorptive desulfurization of thiophenic sulfur compounds using nitrogen modified graphene. *Sep. Purif. Technol.* **2024**, *331*, 125693.
52. Zhao, Z.; Zuhra, Z.; Qin, L.; Zhou, Y.; Zhang, L.; Tang, F.; Mu, C. Confinement of microporous MOF-74(Ni) within mesoporous γ-Al_2O_3 beads for excellent ultra-deep and selective adsorptive desulfurization performance. *Fuel Process. Technol.* **2018**, 176, 276-282.

https://doi.org/10.3390/gels12010033
